# Supplementary material for: An enzyme activation network reveals extensive regulatory crosstalk between metabolic pathways
Source: Mol Syst Biol. 2025 May 22;21(7):870–88. doi: 10.1038/s44320-025-00111-7 (PMC12222706; doi:10.1038/s44320-025-00111-7)
Supplement: Supplementary file 3 — Expanded View Figures [file 44320_2025_111_MOESM3_ESM.pdf]

Expanded View Figures

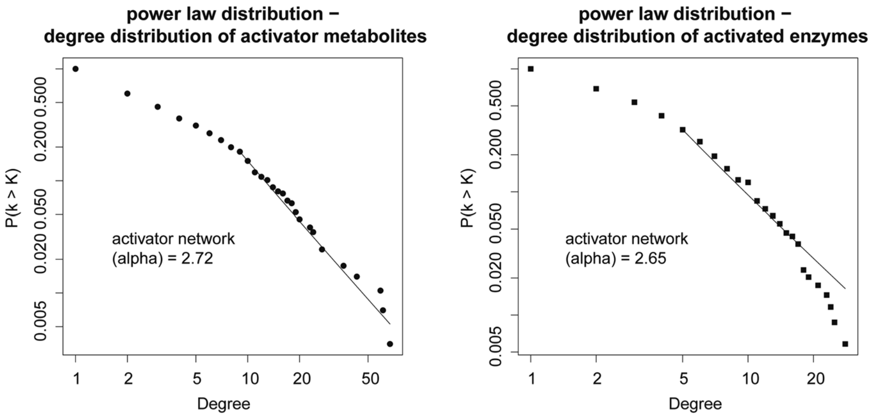

Figure EV1. Degree distribution of nodes in the activation network.

Degree distribution of activator metabolites and activated enzymes in the cell-intrinsic activation interaction network follow power law distribution.

© The Author(s)

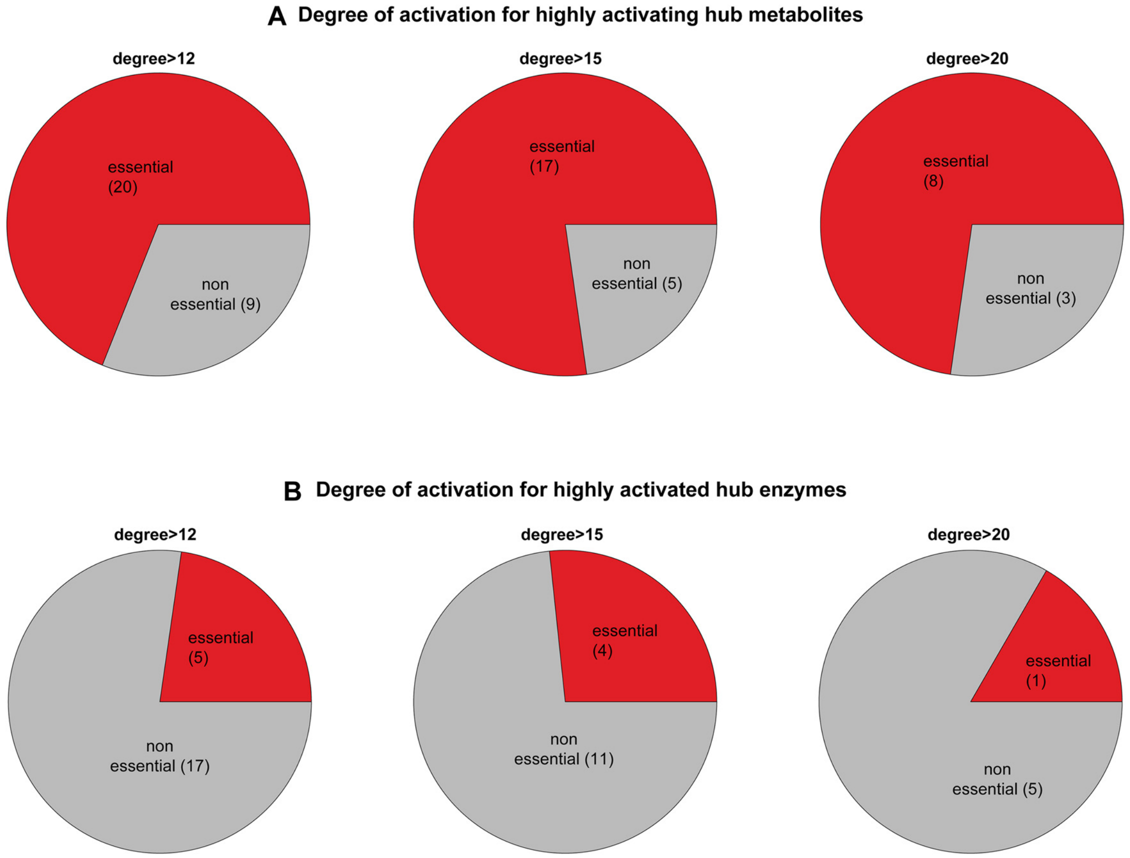

Figure EV2. Essentiality of highly connected activators and enzymes.

(**A**) The degree distribution of activator metabolites within the cell-intrinsic activation interaction network reveals that highly interactive activators (degree > 12, degree > 15, degree > 20) are mostly essential for growth. (**B**) The degree distribution of activated enzymes within the cell-intrinsic activation interaction network reveals that highly activated enzymes (degree > 12, degree > 15, degree > 20) are mostly non-essential for growth.

 © The Author(s)

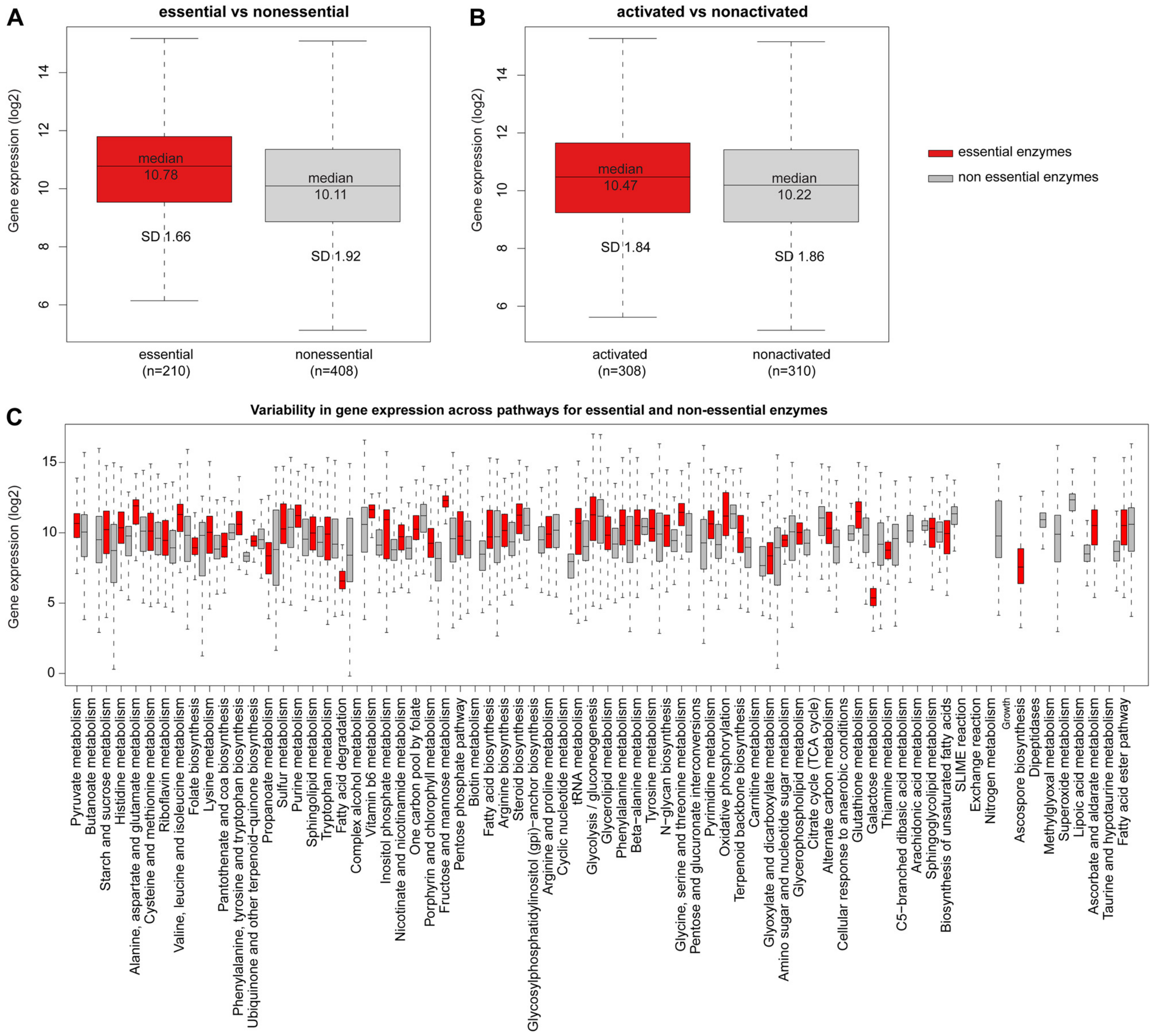

Figure EV3. Variation in gene expression across different enzyme groups.

Gene expression variation among (**A**) essential vs non-essential enzymes, and (**B**) activated vs non-activated enzymes. (**C**) Pathway wise gene expression variation between essential and non-essential enzymes: For each metabolic pathway, a set of essential and non-essential enzymes were identified using in silico enzyme knockout experiments and gene expression variation between essential and non-essential enzymes was calculated using the public data obtained from microarray gene expression experiments. Box plots display the median, the upper and lower quartiles, and the minimum and maximum values represented by the whiskers.

© The Author(s)

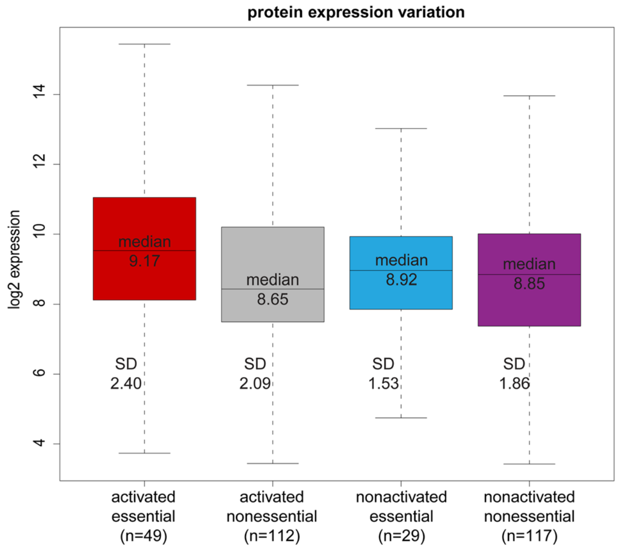

Figure EV4. The protein expression variation among activated essential enzymes, activated non-essential enzymes, non-activated essential enzymes, and non-activated non-essential enzymes.

Box plots display the median, the upper and lower quartiles, and the minimum and maximum values represented by the whiskers.

© The Author(s)

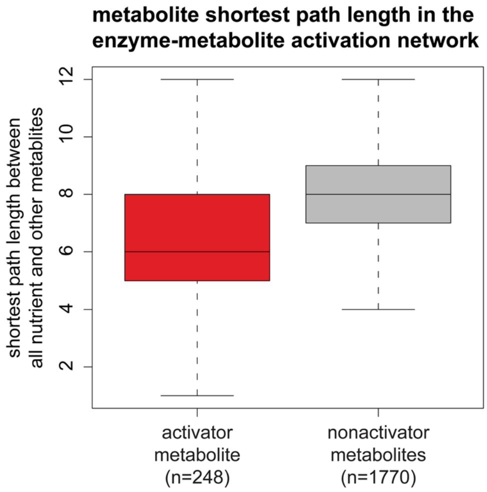

Figure EV5. Shortest path length of activator and non-activator metabolites within the cell-intrinsic activation interactions network from all nutrient compounds.

Box plots display the median, the upper and lower quartiles, and the minimum and maximum values represented by the whiskers.
